# Double burden of malnutrition: A silent driver of double burden of disease in low– and middle–income countries

**DOI:** 10.7189/jogh.02.020303

**Published:** 2012-12

**Authors:** Ivana Kolčić

**Affiliations:** Croatian Centre for Global Health, University of Split School of Medicine, Split, Croatia

Double burden of diseases in low– and middle–income countries (LMICs) is well recognised. However, proper understanding of the need for a joint intervention against both infectious diseases and non–communicable diseases (NCD) has arisen only recently [1]. In 2008, the proportion of premature deaths due to NCD in population under 60 years of age in low–income countries was 41%, in lower middle–income countries 28%, and in high–income countries only 13% [2]. The most frequent causes of death included cardiovascular diseases, diabetes, cancers and chronic lung disease, and the main underlying risk factors were increased blood pressure (responsible for 13% of deaths globally), tobacco use (9%), elevated blood glucose levels (6%), physical inactivity (6%), and overweight and obesity (5%) [3]. Excessive intake of calories is one of the main common factors behind those conditions and risk factors, along with other lifestyle choices and genetic predisposition.

On the other hand, communicable diseases are still difficult to control, especially in young children, even though most of the necessary tools and knowledge about their prevention, treatment and control are available [4]. Those tools are both effective and affordable, but they do not reach those who need them [5]. Four communicable diseases still account for nearly 50% of global child mortality – acute respiratory diseases, diarrhoea, neonatal sepsis and malaria [6]. An important underlying risk factor for those diseases is undernutrition. It was estimated that as much as 35% of child deaths could be attributed to macro– and micro–nutrient undernutrition [7]. In addition to its effect on mortality, undernutrition also affects human development in many aspects.

Recently, it became increasingly apparent that, in addition to ‘double burden of disease’ affecting LMIC populations, there is also ‘double burden of malnutrition’, consisting of undernutrition among children and overnutrition in adults. A driving force behind the shift from undernutrition in childhood to overnutrition in adulthood in LMIC was the rapid increase in economic development, globalization, and urbanization, leading to tremendous changes in lifestyle marked predominantly by changes in diet and physical activity and under– and overnutrition occurring simultaneously among different population groups. This was recognised recently in FAO's document on double burden of malnutrition in six LMIC: China, Egypt, India, Mexico, the Philippines and South Africa [8]. Great disparities were observed: in the Philippines, 27% of children under five years of age were underweight, while 27% percent of women were overweight or obese [8].

Examples of simultaneous occurrence of undernutrition in deprived parts of the population and obesity among more affluent were well recognized in many countries, but these recent changes tend to result in the opposite manifestations of malnutrition even within a single household. For instance, an underweight child and an overweight mother within the same household were observed in 11% of the households in rural areas in Indonesia and 4% in Bangladesh [9]. The figures were even worse in the refugee population living in Western Sahara, in a protracted emergency and dependent on food assistance, where 24.7% of pairs of children aged 6–59 months and mothers aged 15–49 years were affected by this ‘double burden of malnutrition’ [10]. Interestingly, in the same study only 2.4% of children were overweight (29.1% were stunted and 18.6% were underweight), while among the women, 53.7% were overweight or obese, and only 14.8% were stunted [10]. Such differences were explained to arise due to the feeding practices and beauty perception of the Sahrawi population, levels of physical (in)activity, conditions within the refugees camps, nutrients available, but also with the emerging evidence of association between childhood undernourishment and the adult obesity [10].

Almost two decades ago Barker proposed his ‘fetal origins hypothesis’, stating that “fetal undernutrition in middle to late gestation, which leads to disproportionate fetal growth, programmes later coronary heart disease” [11]. His hypothesis was based on ecological studies, but it was soon confirmed in experimental animal models [12,13] and numerous epidemiological studies in different human populations [14-17]. The basic idea underlying this hypothesis was ‘developmental plasticity’: the phenomenon that enables the development of different end–results within a single organism, given the current environmental conditions; those can range from under–representation of important inputs (such as nutrients), to their over–representation. Sometimes, even the same detrimental effects can arise, but through different adaptation mechanisms, like in the case of obesity and type 2 diabetes [18]. Barker's theory laid the foundations for the developmental model of the origins of the adult diseases, namely for NCD, through programming mechanisms including particular cellular signalling pathways, metabolic and hormonal responses, but also through certain epigenetic modifications [19] which bring permanent changes, leading to disease manifestation in the adulthood. Such ‘metabolic programming’ has been so far described for obesity [20,21], type 2 diabetes [18], hypertension [22] and other cardiovascular diseases [23], but also for some other diseases, like asthma [24] and lung cancer [25]. There are also some conflicting results, such as the lack of apparent association between low birth weight and adverse effects on health among children and adults. Also, some studies used less reliable study designs, such as ecological, cross–sectional or case–control designs. In order to gain more accurate and credible findings, a life–course approach to the investigation of environmental, metabolic and genetic factors is necessary and warranted [26]. This approach is defined as “the study of long–term effects on chronic disease risk of physical and social exposures during gestation, childhood, adolescence, young adulthood and later adult life” [27].

**Figure:**
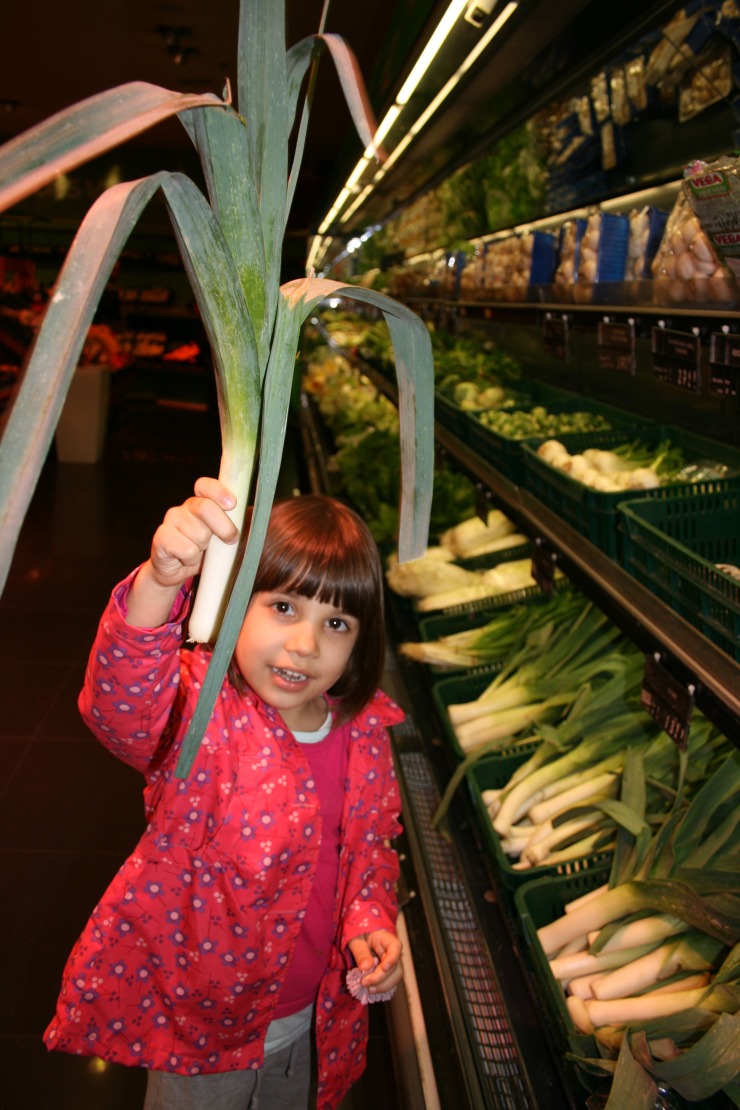
Photo: courtesy of Ivana Kolčić, personal collection

Fetal origins hypothesis is also known as ‘thrifty phenotype’ hypothesis, as opposed to ‘thrifty genotype’ hypothesis, which should also be mentioned here. History of modern humans, dating to the last 120 000 years, was characterised primarily with long periods of food insecurity and scarce resources among hunter–gatherer populations, and only occasional and short–lasting circumstances of abundance. Due to such environmental conditions, early people developed thrifty genotype, enabling them to survive starvation periods and to maximally harness rare opportunities of abundance, storing the energy for upcoming famine. In modern times, dramatic changes resulting in increased food availability led to predisposition to obesity and diabetes type 2 [28].

**Figure:**
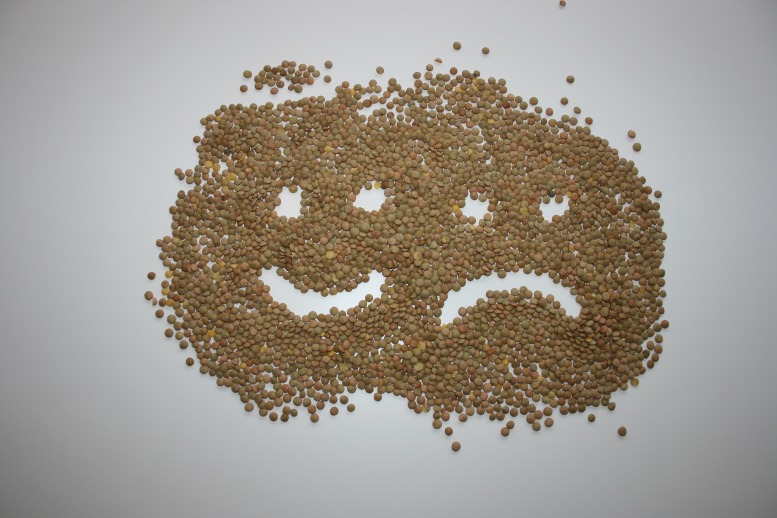
Photo: courtesy of Ivana Kolčić, personal collection

Today's food abundance in high–income countries is marked with loss of seasonality for almost all foods and many fashionable movements in dietary practices, like the ‘Zone diet’, the ‘raw food diet’, the ‘Paleo diet’, the ‘Atkins diet’, the ‘Cactus diet’, the ‘blood type diets’ and many more. Some of those diets seem to be based on unbalanced nutrients and may even generate opposite effects from those desired. Scientific evidence for the effectiveness and appropriateness of any of those diets is insufficient at best.

Scientific inquiry in food and nutrition practices and their effects is well established, which is perhaps best noted in 63 different journals referenced in the National Center for Biotechnology Information Databases (NCBI) having the word ‘food’ or ‘nutrition’ in the journal’s name. A review of the research focused on food safety and security during the last decade showed a steadily growing interest and almost 40–fold increase in the number of citations of the published papers between 2000 and 2010 [29].

The analysis of the scientific literature on adult obesity and cardio–metabolic risk revealed a U–shaped correlation between the birth weight and the risk for the disease outcome [17]. This means that both low birth weight (which can be due to maternal undernutrition) and high birth weight (due to maternal obesity and/or gestational diabetes) are associated with greater risk for adverse outcomes in offspring – particularly adult obesity and diabetes. Intrauterine growth restriction (IUGR) permanently alters fetal metabolism to enable survival in restricted environment. When a child with IUGR is born and raised in the environment rich in high–calories diet, which are becoming more frequent in middle–income and even in low–income countries, it will likely become obese. On the other side of the spectrum, energy–rich environment during foetal life, which is due to maternal overnutrition, may drive the development of excess abdominal fat and type 2 diabetes in later life [18]. This problem is concerning on the broader population level, given that obesity pandemic is a relatively recent phenomenon, leading to high (and still rising) prevalence of overweight and obesity in women in reproductive age around the world – in both developed and in developing countries. If epigenetic mechanisms indeed enhance propensity to adiposity through mechanisms proposed above, the human population may have entered a spiral that will make each new generation more obese. For instance, according to the results on national, regional, and global trends in body–mass index (BMI) since 1980, BMI increased by 0.5 kg/m^2^ per decade in women worldwide and by 0.4 kg/m^2^ per decade for men [30]. More than half of the adults in high–income countries and in upper middle–income countries were overweight, but in lower middle– and low–income countries the increase in prevalence of overweight and obesity over the last three decades was greater than in upper middle– and high–income countries [30].

There is ample scientific evidence on the effects of overweight and obesity on health, ranging from local tissue inflammation to atherosclerosis, myocardial infarction, diabetes, hypertension, hyperlipidemia, some forms of cancer, locomotor problems, gout, urinary stones formation, gallbladder disease, sleep disorders, excessive sweating, and others. Obese people also suffer from social stigmatization and isolation, which can easily lead to depression [31] and overall poor quality of life. Furthermore, people with obesity have shorter life span and an increased risk of sudden death [32]. Therefore, obesity is the crucial mediator between the unhealthy lifestyle, marked by the unhealthy dietary patterns, coupled with poor physical activity, and morbidity and mortality from many non–communicable chronic diseases.

Poverty remains the most important reason for stunting and wasting, which are the most commonly used indicators of malnutrition among children. However, recent findings from the USA described the link between poverty and obesity, mediated through affordability of unhealthy foods. Obesity in North America is significantly more prevalent in poor neighbourhoods and among groups with lower education and income, suggesting inequitable access to healthy foods. This trend is mainly driven by the prevalent consumption of grains, added sugars and fats, which are inexpensive, good–tasting, convenient and low–cost [33]. In the survey among US adults in 12 states during 2009, those who felt insecure about food availability had 32% increased odds of being obese compared to those who felt secure [34]. These findings suggest the presence of the burden of undernutrition within the burden of energy overnutrition. Continuing global recession and economic downfall is likely to further aggravate those negative trends in the future.

The burden of malnutrition is enormous. In 2009, over a billion people were reported as food insecure and 180 million children were reported being undernourished [35]. At the same time, the global estimation from 2008 amounts for 1.4 billion overweight adults and 40 million overweight children, with over 200 million obese men and 300 million obese women [36]. There is no easy solution to overcoming this perplexing problem, but successful strategies will need to incorporate increased reliance on local resources through integrative approaches in all the countries of the world [35].

Described mechanisms and trends highlight a ‘double burden of malnutrition’ as an important driver of the double burden of disease. On one hand, undernutrition in fetal life and among children predisposes to infectious diseases, but also increases the NCD burden, mainly through overweight and obesity and related co–morbidities. On the other hand, overnutrition in pregnant overweight women closes the circle. Since the abundance of literature is supporting these findings in developed countries, the real question is what the future holds for the developing countries? This problem should be given greater attention, so that a scenario for the future of mankind that was very intelligently portrayed in WALL–E movie is avoided [37].
